# A Comparison of Nurse-Driven and Combined Physician and Nurse-Driven Interventions for Improving Sleep Quality and Quantity in Hospitalized Patients

**DOI:** 10.7759/cureus.111395

**Published:** 2026-06-23

**Authors:** Mitchell T Wong, Alexander T Lo, Stephanie D Abdulrahman, Reid Sasaki, Gregory Seymann, Alan Moazzam

**Affiliations:** 1 Internal Medicine, UC (University of California) San Diego School of Medicine, San Diego, USA; 2 Medicine, UC (University of California) San Diego School of Medicine, San Diego, USA; 3 Nursing, University of California, San Diego Health, San Diego, USA; 4 Hospital Medicine, University of California, San Diego Health, San Diego, USA

**Keywords:** inpatient sleep quality, patient satisfaction, sleep in hospitalized patients, sleep interventions, sleep promotion

## Abstract

Background

Inpatient sleep disruption is prevalent and contributes to delirium, impaired recovery, and increased healthcare utilization. Causative factors include illness severity, unfamiliar surroundings, circadian disruption, and poorly timed clinical tasks. Non-pharmacologic, protocol-driven approaches to improving inpatient sleep remain underutilized despite evidence of their benefit. This study aimed to evaluate the effectiveness of nurse-driven and combined nurse-physician sleep interventions in subjective sleep quality, patient satisfaction, and hospital resource utilization among medical-surgical inpatients.

Methods

Three sequential cohorts of medical-surgical inpatients were enrolled: a control group receiving standard care; a nurse-intervention cohort receiving nightly nurse-led sleep rounds and a personalized Sleep Hygiene Menu encompassing environmental modifications, comfort measures, and relaxation aids; and a combined nurse-physician cohort in which hospitalists additionally modified overnight order sets to reduce non-urgent vital sign checks, laboratory draws, and medication administrations during a protected sleep window (10:00 PM-6:00 AM). Cohorts were compared on demographics, LACE index scores (an acronym for Length of stay, Acuity of admission, Comorbidity, and Emergency department visits), responses to a modified Richards-Campbell Sleep Questionnaire (RCSQ), and Delirium Observation Screening Scale (DOSS) scores. Hospital Consumer Assessment of Healthcare Providers and Systems (HCAHPS) scores and sitter hours were captured for the control and nurse-intervention cohorts.

Results

A total of 92 patients were enrolled (29 control, 31 nurse intervention, 32 nurse+physician intervention), generating 102 survey responses (38 control, 32 nurse intervention, 32 nurse+physician intervention). Both intervention cohorts reported significantly deeper sleep, shorter sleep latency, fewer nocturnal awakenings, faster return to sleep, and better overall sleep quality compared to controls (all p < 0.05). No significant differences were found between the two intervention groups (p > 0.05 for all values). HCAHPS data showed an 8.47% increase in patient satisfaction with nighttime quietness (p = 0.0007). Sitter hours were reduced by 896 hours over seven months, yielding $21,351.68 in cost savings.

Conclusion

Structured nurse-driven sleep interventions significantly improved inpatient sleep quality and patient satisfaction, and generated measurable cost savings. Adjunctive physician order modifications provided no additional benefit over nursing interventions alone. These findings support routine integration of sleep hygiene protocols into inpatient nursing care and highlight an opportunity for hospitalists to champion multidisciplinary sleep promotion as a quality and cost-reduction strategy. Future studies should incorporate objective sleep measurement using wearable actigraphy devices.

## Introduction

Hospitalized patients experience poor sleep and frequently receive hypnotic medications as a consequence [1,2]. Inadequate, interrupted sleep disrupts circadian rhythms and adversely affects clinical outcomes. Inpatient sleep deprivation is associated with reduced patient satisfaction and Hospital Consumer Assessment of Healthcare Providers and Systems (HCAHPS) scores [3], higher rates of delirium, increased use of sedative-hypnotic agents, prolonged length of stay, worsened medical outcomes, and greater hospital resource utilization [4-6]. A structured sleep protocol comprising a bundle of nurse- and physician-driven interventions, anchored by nightly nurse-led sleep rounds, was implemented to reduce unnecessary overnight interruptions and promote a hospital environment conducive to restorative sleep [7-9].

This pilot study evaluated the impact of a bundle of sleep-promoting nursing and combined nursing-physician interventions on subjective and objective outcomes among eligible medical-surgical inpatients.

## Materials and methods

Patient selection and data collection

Three sequential cohorts of patients were enrolled from a single medical-surgical unit at UC San Diego Jacobs Medical Center. The first cohort served as a control group and received standard care from November 2021 to January 2022. The second cohort received nurse-driven sleep interventions from March 2022 to May 2022. The third cohort received combined nursing and physician interventions from November 2023 to May 2024. Cohort demographics are shown in Table 1.

**Table 1:** Demographics of patients in the control and two intervention groups Patient demographics and baseline clinical metrics are shown for each of the three sequential cohorts. Age is reported as mean (range). Gender is reported as the male-to-female ratio. The LACE index (Length of stay, Acuity of admission, Comorbidity, and Emergency department visits) [10] scores and Delirium Observation Screening Scale (DOSS) [11] scores are reported as cohort means. No statistically significant differences in age or LACE scores were identified between the intervention cohorts and the control group (p > 0.05 for all comparisons), confirming baseline comparability across study periods.

	Control	Nurse intervention	Nurse + physician intervention
Number of patients	29	31	32
Number of surveys	38	32	32
Gender (M:F)	19:19	20:12	16:16
Age (range)	56 (21-81)	55 (23-88)	54 (24-79)
Average LACE score	64	60	67
Average DOSS score	0.31	0	0

Before implementation, the protocol was formally introduced to nursing staff at a Unit-Based Practice Council meeting, where study aims, methodology, and data collection procedures were presented. A two-week in-service education period followed, during which staff received in-person training on the rationale for the sleep hygiene protocol, implementation steps, and documentation expectations. Staff competency was validated through a structured sign-off process before the intervention phase commenced. During the pre-intervention (control) phase, patients were randomly approached each morning and invited to complete a questionnaire regarding their sleep experience during hospitalization. During the implementation phases, patients admitted to the unit who met the eligibility criteria were introduced to the Sleep Hygiene Menu by nursing staff and invited to participate in the sleep study.

Sleep quality was assessed using a modified version of the Richards-Campbell Sleep Questionnaire (RCSQ) [12], a validated, five-item instrument that evaluates sleep depth, sleep latency, nocturnal awakenings, ability to return to sleep, and overall sleep quality, with an optional sixth item assessing perceived nighttime noise. In its original form, each RCSQ item is rated using a 100-mm visual analog scale anchored by opposing descriptive statements (e.g., "light sleep" versus "deep sleep"). In the present study, response options were simplified to a binary choice between the two anchor statements for each domain to reduce respondent burden in the acute inpatient setting. Delirium risk was assessed using the Delirium Observation Screening Scale (DOSS) [11], a validated, nurse-administered bedside screening tool for detecting delirium in hospitalized patients. Approval for use of the modified RCSQ was obtained from Dr. Kathy C. Richards, the original developer of the instrument.

**Figure 1:**
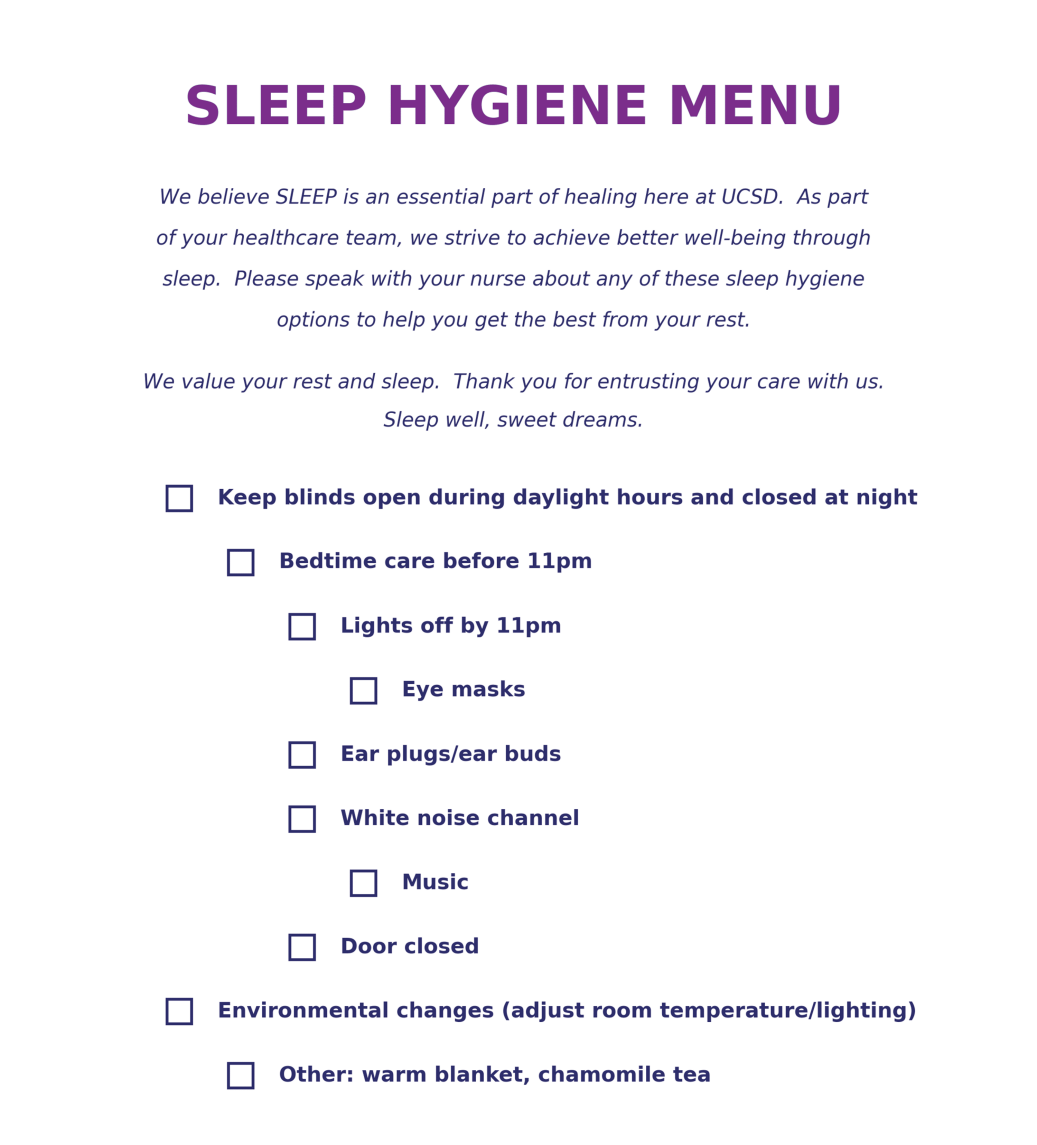
Sleep Hygiene Menu for sleep intervention cohort patients This Sleep Hygiene Menu was developed and designed by the study team at the Department of Hospital Medicine, UC San Diego Health, for use in this quality improvement project. The menu represents standard institutional practice material created for patient-facing use on the study unit. The menu was created using Microsoft Word (Microsoft Corporation, Redmond, USA).

The purpose of the questionnaire and study procedures was explained before participation. Upon verbal agreement to participate, participants were informed that their responses would be collected and analyzed to evaluate potential improvements in sleep quality associated with the Sleep Hygiene Protocol. Formal written informed consent was not required; the ACQUIRE (Aligning and Coordinating Quality Improvement, Research, and Evaluation) approval committee waived consent on the basis that the study aimed to modify nursing practices rather than the standard of care. Patients were enrolled from a convenience sample hospitalized on the specified medical-surgical unit during each study period. Specific eligibility criteria were established to ensure patient safety and the appropriateness of interventions. Eligible participants were required to be alert and oriented to person, place, time, and situation (A&O×4), functionally independent, and clinically stable at a medical-surgical level of care. Patients were excluded if they required frequent overnight monitoring, including recurrent vital sign assessments, laboratory draws, or overnight medication administration, which would preclude uninterrupted sleep. Additional exclusion criteria included the need for respiratory support, such as continuous positive airway pressure (CPAP), and non-English-speaking status. These criteria were established to ensure that sleep hygiene interventions could be implemented safely without compromising necessary clinical care. For the nursing intervention arm, nurses administered a study-generated Sleep Hygiene Menu (Figure 1) to identify each patient’s preferred sleep interventions. Patients in the control arm received no interventions and completed only sleep quality questionnaires. For the combined nursing and physician intervention arm, hospitalists modified non-urgent overnight orders at the time of patient enrollment, in addition to all nursing interventions. Order modifications targeted routine vital sign frequency, laboratory draw timing, and non-time-critical medication scheduling to protect the core sleep window (10:00 PM-6:00 AM); medically necessary orders were not altered. Sleep hygiene interventions were maintained nightly throughout each patient’s admission unless clinical status changed. In patients who became hemodynamically unstable or required transfer to a higher level of care, sleep hygiene measures were suspended due to the clinical need for increased overnight monitoring.

Outcome metrics collected across all cohorts included demographics (age and sex), LACE index scores (a validated composite measure of length of stay (L), acuity of admission (A), comorbidity burden (C), and prior emergency department visits (E)) [10], responses to the modified RCSQ and DOSS scores [11], and length of stay. Permission to use the LACE index was obtained from Dr. Carl van Walraven, the original developer of the instrument.

HCAHPS scores and sitter hours were captured for the control and nurse-intervention cohorts to quantify patient satisfaction and cost impact. HCAHPS 'quiet at night' responses were dichotomized for analysis using the Centers for Medicare & Medicaid Services (CMS) top-box scoring methodology, with 'Always' categorized as quiet and all other responses ('Never,' 'Sometimes,' or 'Usually') categorized as not quiet. A chi-square test of independence was applied to HCAHPS data to assess differences before and after the nursing intervention.

Description of nursing interventions

All nursing interventions were documented in the electronic health record. Upon enrollment, each patient in the nursing intervention cohort was presented with a personalized Sleep Hygiene Menu (Figure 1), a study-generated checklist of evidence-based, non-pharmacologic sleep-promoting options from which patients selected preferred strategies. The menu encompassed three categories: (1) environmental modifications, including dimming room lights by 10:00 PM, closing the room door fully, applying window blinds to block external light, and offering a sleep mask and earplugs; (2) comfort measures, such as providing an additional blanket, a warm non-caffeinated beverage before sleep, and room temperature adjustment; and (3) relaxation aids, including white noise or nature sounds, and patient-directed music or audio. Nightly “sleep rounds” were conducted between 9:00 PM and 10:00 PM to confirm patient preferences, initiate interventions, and address barriers to sleep. Selected interventions were maintained throughout each patient’s admission and reassessed nightly; measures were suspended only if clinical status deteriorated to a degree requiring increased overnight monitoring. Sleep quality was assessed weekly (every Friday morning) by registered nurses using the Sleep Hygiene & Well-Being Survey (modified RCSQ); patients discharged before seven days were assessed on the day of discharge.

Description of physician interventions

For the combined cohort, hospitalists entered protocol-based order modifications at the time of enrollment, applied in addition to all nursing interventions. Three categories of routine overnight orders were targeted: (1) in terms of vital sign frequency for hemodynamically stable patients without active deterioration, routine overnight checks were reduced from every four hours to every eight hours or as needed between 10:00 PM and 6:00 AM; (2) laboratory draw timings of non-urgent studies (e.g., routine complete blood counts, metabolic panels) were shifted from overnight or early-morning collection to mid-morning draws after 8:00 AM, provided results were not required for immediate clinical decisions; and (3) medication scheduling for non-time-critical medications (e.g., once-daily vitamins, scheduled laxatives, elective antihistamines) was consolidated into daytime windows. Clinically necessary orders, including insulin sliding-scale dosing, scheduled antiemetics, PRN (pro re nata) analgesics, and telemetry protocols, were preserved without modification. Eligibility for order modifications was confirmed during the daytime rounding visit and documented in the progress note. Physician interventions were suspended for any patient whose clinical status deteriorated to the point of requiring increased overnight monitoring.

Data analysis

Cohorts were compared on demographics (age and sex), LACE scores, RCSQ responses, and DOSS scores. Independent-samples t-tests were used to assess between-cohort differences in age and LACE score distributions, quantifying baseline morbidity comparability across groups. Chi-square tests of independence were applied to RCSQ responses to evaluate whether sleep intervention assignment was associated with differences in reported sleep outcomes. Cohorts did not differ significantly in age or LACE scores (p > 0.05 for all comparisons), indicating adequate baseline comparability.

## Results

Responses to the RCSQ domains of sleep depth, sleep latency, nocturnal awakenings, return to sleep, overall sleep quality, and perceived noise level were recorded for each cohort (control, n=38; nurse intervention, n=32; nurse-physician intervention, n=32). Questionnaire results are summarized in Table 2.

**Table 2:** Sleep quality survey results for control and the two intervention cohorts The Richards-Campbell Sleep Questionnaire used in this study was modified to provide participants with two answer choices for each category. The raw number of responses for each category is recorded, along with the percentage proportions in each group. With the exception of ‘noise level,’ all responses between the control and two intervention groups were statistically significant (p < 0.05). χ​​​​​​​^2^(1) is the chi-square statistic, and Cramér's V is the Cramér's V effect size. *Statistically significant.

Richards-Campbell Sleep Questionnaire	Control (no sleep interventions), November 2021–January 2022		Nursing intervention, March 2022–May 2022		Nursing + physician intervention, November 2023–May 2024		p-values for control-nursing interventions, χ^2^(1), Cramér's V	p-values for control-nursing + physician interventions, χ^2^(1), Cramér's V
	n=38	%	n=32	%	n=32	%		
Sleep depth							0.0056*, 7.68, 0.33	0.0496*, 3.86, 0.24
Light	22	57.90	8	25.00	11	34.38		
Deep	16	42.10	24	75.00	21	65.62		
Sleep latency							0.0003*, 13.02, 0.43	0.0041*, 8.24, 0.34
Delay	15	39.50	1	3.10	3	9.38		
Fast	23	60.50	31	96.90	29	90.62		
Awakenings							0.0079*, 7.05, 0.32	0.0079*, 7.05, 0.32
All night	10	26.30	1	3.10	1	3.12		
Few	28	73.70	31	96.90	31	96.88		
Return to sleep							0.0002*, 13.44, 0.44	0.0137*, 6.08, 0.30
Slow	13	34.20	0	0	3	9.38		
Fast	25	65.80	32	100	29	90.62		
Sleep quality							0.0005*, 12.20, 0.42	0.0005*, 12.20, 0.42
Bad	12	31.60	0	0	0	0		
Good	26	68.40	32	100	32	100		
Noise level							0.231, 1.44, 0.14	0.0587, 3.57, 0.23
Noisy	4	10.50	1	3.10	0	0		
Quiet	34	89.50	31	96.90	32	100		

Age and LACE scores did not differ significantly between either intervention cohort and the control group (p > 0.05 for all comparisons), confirming baseline comparability. All RCSQ domains, except perceived noise level, differed significantly between the control cohort and each intervention cohort (Table 2). Both intervention cohorts reported improvements in sleep depth, latency, nocturnal awakenings, return to sleep, and overall sleep quality compared with controls, with effect sizes ranging from small to moderate (Cramér's V = 0.24-0.44). Perceived noise level did not differ significantly between controls and the nurse-only cohort, though a trend toward significance was observed for the nurse-physician cohort. No significant differences in any RCSQ domain were identified between the nurse-only and nurse-physician cohorts (p > 0.05 for all comparisons, n=64). Full pairwise chi-square statistics and effect sizes for all comparisons are presented in Table 2.

**Figure 2:**
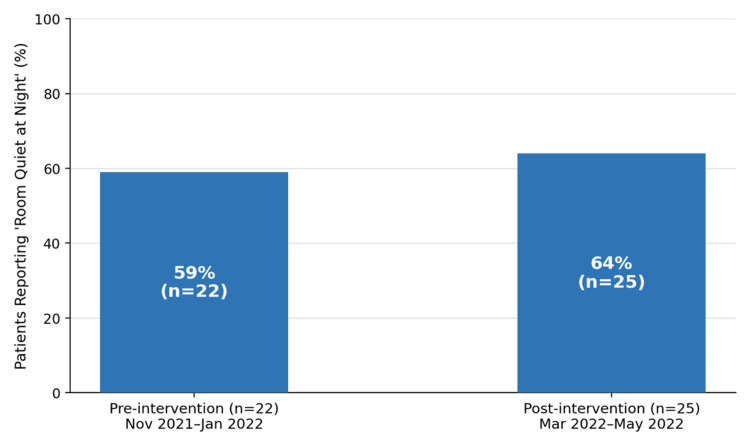
Hospital Consumer Assessment of Healthcare Providers and Systems (HCAHPS) survey results for pre- and post-nursing interventions Frequency (n) and percentage of patients reporting the room as quiet at night are shown within each bar. Baseline data revealed that pre-intervention, 59% of patients in the unit reported, “quiet around the room at night.” Three months post-intervention, 64% of patient participants reported, “quiet around the room at night.”

HCAHPS data demonstrated an 8.47% increase in patient-reported nighttime room quietness following the implementation of nursing interventions (p=0.0007; Figure 2).

**Figure 3:**
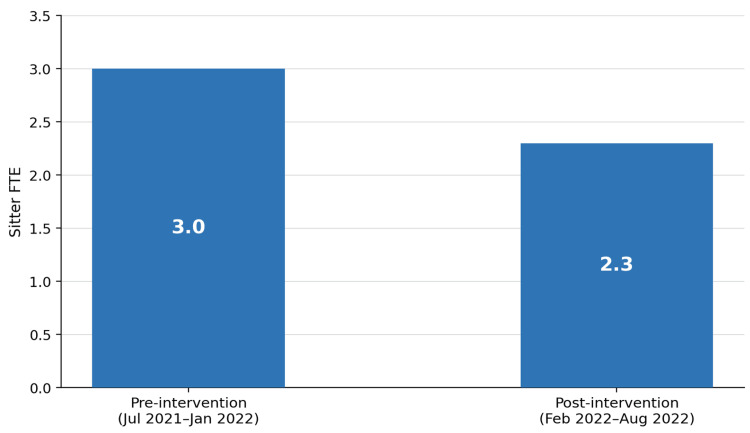
Sitter hours full-time equivalent (FTE) for the pre-nursing intervention (July 2021-January 2022) versus post-nursing intervention (February 2022-August 2022) One sitter FTE equals 1280 hours. For the pre-nursing intervention, 3 sitter FTE = 3840 hours. For the post-nursing intervention, 2.3 sitter FTE = 2944 hours. Difference: 3840 – 2944 = 896 hours (reduced sitter hours). Given the senior nursing aide's average hourly salary is $23.83, $23.83 x 896 hours = $21,351.68 total cost savings from nursing sleep interventions over seven months.

Sitter utilization decreased by 896 hours over the seven-month post-intervention period compared with the pre-intervention period, yielding estimated cost savings of $21,351.68 (Figure 3). HCAHPS scores and sitter hours were not captured for the combined nursing-physician intervention period.

## Discussion

Nurse-driven and combined nurse-physician sleep interventions were associated with significant improvements in inpatient sleep quality across multiple domains, consistent with prior evidence that modifiable environmental and workflow factors contribute meaningfully to sleep disruption during hospitalization [13,14]. Both intervention cohorts reported superior outcomes compared with controls across nearly all RCSQ domains. Notably, no significant differences were observed between the nursing-only and combined cohorts, suggesting that the benefit of physician order modifications was negligible compared with that provided by nursing interventions alone.

Illness severity represents a principal confounder in inpatient sleep research. Patients admitted with acute pain, dyspnea, or other active symptoms are at heightened risk of poor sleep independent of environmental interventions [15]. To address this, LACE scores were calculated for each cohort as a composite measure of morbidity; between-group comparisons were not statistically significant (p=0.256), supporting comparability across study periods.

Despite improvements across all other sleep domains, perceived noise level did not differ significantly between groups (p=0.231, Table 2). Several interventions in the nursing protocol were implemented to attenuate the subjective impact of ambient noise and directly alter noise levels, including the use of earplugs, white-noise devices, and door closure [16]. However, the degree of attenuation was insufficient for patients to mark “very quiet” on the survey, and it is possible that patients who felt the environment was somewhat noisy selected the closest option “very noisy”. Although it is difficult to make the hospital environment as quiet as possible for patients, our results indicate that patient-centered sound-masking strategies can meaningfully mitigate noise-related sleep disruption, as reflected in the interventional groups’ significantly favorable responses to the other RCSQ questions.

Self-reported sleep measures are subject to response bias, as participants may rate their sleep quality higher due to social desirability or inaccurate recall. The lack of significant differences between the nursing and nursing + physician intervention groups (p > 0.05 for all values) suggests that the studied physician interventions of rescheduling vital signs, lab draws, and medication administrations to protect sleeping hours do not provide additional benefit in patients’ perceptions of their sleep quality, compared to when only nursing sleep interventions are applied. This result suggests that nursing interventions yield sleep-improvement benefits that exceed those of physician interventions for sleep quality. This may be because nurses interface with patients more frequently than physicians and can improve the sleep environment to a higher degree during the patient’s hospitalization, whereas vital signs, lab draws, and exams occur periodically during the day.

Nurse-driven sleep protocols are among the most practical and cost-effective interventions available to inpatient care teams. Implementation was associated with an 8.47% increase in HCAHPS nighttime quietness scores (p=0.0007) and a reduction of 896 sitter hours over seven months, yielding $21,351.68 in cost savings. This reduction in sitter utilization may reflect a decrease in hospital-acquired delirium attributable to improved sleep quality, given the established relationship between sleep deprivation and delirium incidence. While this association is correlational, it underscores the potential for sleep promotion protocols to generate measurable financial returns alongside clinical benefits.

These findings carry direct implications for hospitalists. Although physician order modifications alone did not produce incremental gains in this study, hospitalists occupy a critical role as champions of sleep-focused care: by formally enrolling patients in sleep protocols, adjusting overnight order sets where clinically safe, and reinforcing sleep hygiene goals during rounds, hospitalists can create the institutional conditions under which sleep interventions thrive.

Light-based interventions, including keeping blinds open during the day and closed at night, completing bedside care before 11:00 PM, and extinguishing room lights at 11:00 PM, do not directly attenuate ambient noise yet were associated with meaningful improvement in sleep quality: 68.4% of control patients reported good overall sleep quality compared with 100% in both intervention cohorts. These measures likely reinforced physiologic circadian cues, promoting more regular sleep-wake cycling in an environment notorious for circadian disruption. Prior work has demonstrated that entraining circadian rhythms through light cues improves sleep in settings with disrupted schedules, such as night-shift work [17]. Analogously, structured light management during hospitalization may reduce the degree of circadian misalignment that compounds illness-related sleep impairment [18].

An umbrella review by Bellon et al. synthesizing 109 studies of nurse-driven interventions to improve inpatient sleep quality concluded that the impact of broad environmental changes on inpatient sleep, such as reducing ambient light and noise, was inconclusive, whereas the use of earplugs and eye masks showed consistent positive effects [19]. These findings are broadly consistent with the present results. No published studies were identified that directly compared the impact of nursing-only versus combined nursing-physician interventions on inpatient sleep quality, making the present study a novel contribution to this literature.

Many sleep hygiene interventions in our study were nurse-driven and can be integrated into routine clinical practice without a formal research infrastructure. Core components, clustering care to minimize nocturnal disruptions, reducing environmental stimuli, and promoting uninterrupted rest, align closely with standard nursing workflows. Bedside nurses can independently adopt these strategies by anticipating patient needs, coordinating overnight task timing, and limiting non-essential contacts during the protected sleep window. With minimal resource requirements and appropriate unit-level awareness, these interventions can be implemented sustainably to enhance patient rest and support recovery across a broad range of inpatient settings.

Study limitations

This study has several limitations. The sample size was small, and recruitment was confined to a single medical-surgical unit at one academic institution, limiting generalizability. The sequential cohort design, with each group enrolled in separate calendar periods spanning two years, introduces the possibility of temporal confounding from unmeasured changes in unit staffing, patient acuity mix, or institutional practice. Sleep quality was assessed solely by self-report, which is susceptible to recall bias and social desirability effects; objective measurement using actigraphy was not employed. The modification of the RCSQ to binary response options, while pragmatically justified, departs from the validated instrument and may limit comparability with the published normative data. Additionally, HCAHPS scores and sitter hours were not captured for the combined nursing-physician cohort, precluding a complete cost-benefit analysis across all three arms.

The sequential cohort design spans an extended time period, as the interval between cohorts reflected the time required to obtain institutional buy-in and secure participation from the necessary clinical stakeholders rather than any planned methodological gap. While enrollment proceeded without delay once physician engagement and approvals were in place, the possibility of temporal confounders, such as changes in staffing, unit policies, or patient population characteristics across cohorts, cannot be fully excluded.

Validation of these findings in larger, multi-site, randomized controlled trials is warranted. Future studies should incorporate objective sleep measurement via wrist actigraphy, which is feasible in acute care settings and provides data on sleep duration, efficiency, and fragmentation that self-report cannot capture. Investigating the effects of sleep protocols across diverse inpatient settings, including medical, surgical, and step-down units, would improve generalizability. The impact of modified physician rounding times and telemedicine overnight coverage models on sleep disruption also merits a dedicated study. Hospitalist-led quality improvement initiatives that incorporate structured sleep protocol adoption, with nursing as the primary implementation arm, represent a high-yield, low-cost target for future intervention research.

Lastly, the specific sleep interventions selected by individual patients, such as eye masks, earplugs, and other comfort items, were not prospectively recorded. This precluded a formal cost-effectiveness analysis and net savings calculation, as the total material costs attributable to the intervention could not be determined. Future studies should incorporate prospective tracking of individual intervention uptake to enable accurate cost accounting and to identify which specific interventions contribute most to sleep quality improvement.

## Conclusions

A structured, nurse-driven sleep protocol significantly improved subjective sleep quality across multiple domains, increased patient satisfaction with nighttime quietness, and was associated with substantial cost savings through reduced sitter utilization. Adjunctive physician order modifications provided little to no incremental benefit over nursing interventions alone, suggesting that the nursing role was the primary driver of inpatient sleep improvement in our study. These findings underscore the importance of hospitalist-nurse collaboration: hospitalists are well-positioned to champion sleep promotion as a quality priority by formally enrolling patients in protocols, supporting overnight order restructuring, and partnering with nursing leadership to embed sleep hygiene practices into standard inpatient care. Adoption of such protocols offers a low-cost, scalable strategy to improve patient experience, reduce delirium risk, and generate measurable institutional cost savings.
